# Recurrent Spontaneous Pneumothorax in a Young Male With Duchenne Muscular Dystrophy Following COVID-19 Infection

**DOI:** 10.7759/cureus.52408

**Published:** 2024-01-16

**Authors:** Pramod Bhattarai, Monika Karki

**Affiliations:** 1 Pulmonary Medicine, Howard University Hospital, Washington, DC, USA; 2 Critical Care Medicine, Larkin Community Hospital Palm Springs Campus, Hialeah, USA; 3 Internal Medicine, Harlem Hospital Center, New York, USA; 4 Internal Medicine, The Brooklyn Hospital Center, Brooklyn, USA

**Keywords:** chronic hypoxic respiratory failure, robotic thoracoscopy, nippv, covid-19, secondary spontaneous pneumothorax, duchenne muscular dystrophy (dmd)

## Abstract

Duchene muscular dystrophy (DMD) is a genetic disorder primarily affecting males. It is characterized by progressive muscle tissue degeneration. Patients with DMD are at an increased risk of respiratory infections, including coronavirus disease 20019 (COVID-19), due to weakened respiratory muscles. We present a case of a young male with DMD who experienced recurrent pneumothorax 10 months after recovering from a COVID-19 infection. The patient required prompt medical intervention, including a chest tube, multiple surgeries, and mechanical pleurodesis. This case highlights the importance of recognizing recurring pneumothorax as a potential complication of COVID-19, particularly in patients with underlying neuromuscular disorders, as it is a medical emergency requiring prompt treatment to prevent respiratory compromise.

## Introduction

Duchene muscular dystrophy (DMD), caused by a mutation in the dystrophin gene and inherited in an X-linked recessive pattern, affects approximately one in every 5,000 male births [1]. This condition leads to progressive muscle weakness, impacting mobility and respiratory function [1]. Patients with DMD are particularly vulnerable to respiratory infections, including coronavirus disease 20019 (COVID-19) pneumonia, due to weakened respiratory muscles and chronic respiratory failure from restrictive lung disease [2]. Spontaneous pneumothorax, first described in 1819, refers to the presence of air in the pleural cavity without traumatic cause and has emerged as a recognized complication of COVID-19 [2]. Initially attributed to invasive ventilation complications such as barotrauma or volume trauma, recent reports indicate its occurrence without such factors [3]. Spontaneous pneumothorax can be primary or secondary, with the latter associated with underlying lung disease or connective tissue related interstitial lung disease [4]. The annual incidence of spontaneous secondary pneumothorax is approximately 6.3 per 100,000 men and 2 per 100,000 women [4]. On the contrary, the ratio is elevated up to 18% among DMD patients [5]. Recognizing symptoms such as dyspnea, chest pain, and hypoxia is crucial for timely intervention.

This article was previously presented as an oral presentation at the 2023 CHEST Annual Scientific Meeting on October 11, 2023.

## Case presentation

**Figure 1:**
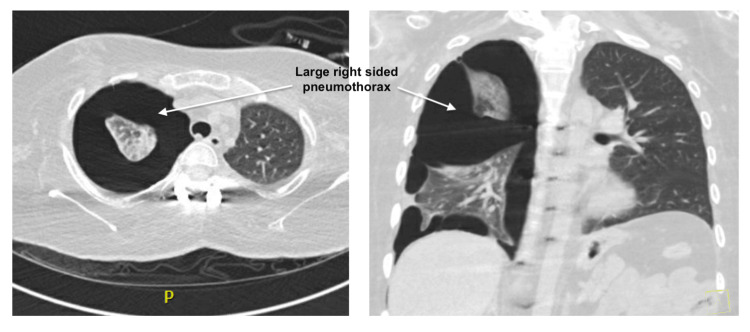
CT of the chest showing a large right-sided pneumothorax with portions of the right lung attached to the lateral chest wall due to adhesions

**Figure 2:**
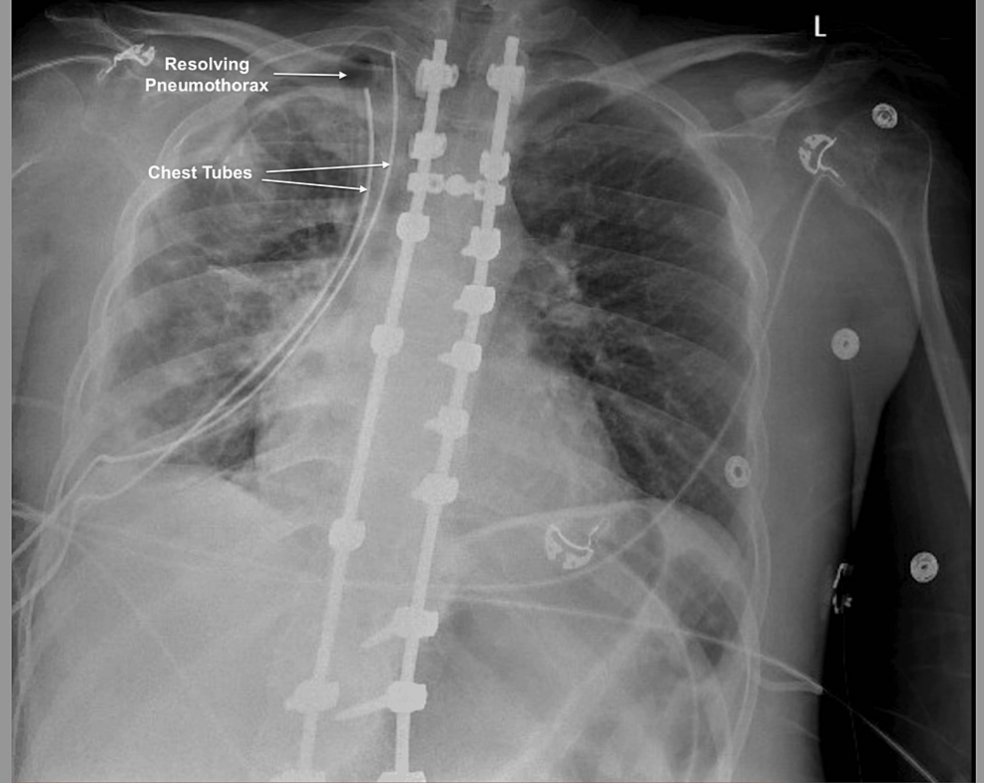
Chest X-ray after chest tube placement with resolving pneumothorax

A 25-year-old-male with a history of DMD, non-invasive positive pressure ventilation (NIPPV) dependent, mild restrictive lung disease, scoliosis with spinal fusion at multiple levels, hypothyroidism, COVID-19 infection seven months prior to presentation, and recent pleurodesis two weeks prior to presentation for spontaneous pneumothorax presented with recurrent right-sided chest pain and shortness of breath. Vital signs were stable, and he was wheelchair-bound, with absent breath sounds on the right side. An electrocardiogram showed normal sinus rhythm with an incomplete right bundle branch block. Chest X-ray revealed a moderate-to-large right-sided pneumothorax with complex features. A subsequent chest CT scan showed a large multi-loculated right-sided pneumothorax (Figure 1), necessitating prompt intervention. After a thorough discussion of the potential risks and benefits of surgery with the patient and his family, they decided to proceed with surgery. He underwent chest tube placement (Figure 2), followed by robotic thoracoscopy, right lobe wedge resection, apical blebectomy, and mechanical pleurodesis, successfully evacuating the pneumothorax. Chest tubes were removed on the post-operative day 6, and he was discharged home on the ninth day with close outpatient follow-up.

## Discussion

DMD, a severe degenerative muscle disease caused by mutations of the skeletal protein dystrophin gene, primarily affects males, with a prevalence of 4.8 cases per 100,000 males worldwide [6,7]. First described in 1857, DMD is characterized by progressive proximal muscle weakness, leading to motor function decline, eventual loss of ambulation, respiratory insufficiency, and cardiomyopathy. Weakened respiratory muscles in DMD patients elevate the risk of respiratory complications, including ineffective cough, nocturnal hypoventilation, and chronic respiratory failure in the setting of underlying restrictive lung disease [6].

The exact mechanism and pathogenesis of spontaneous pneumothorax, a known complication of DMD, are not fully understood [7]. Factors such as subpleural blebs, bullae, emphysema-like changes, and visceral pleural porosity, as well as environmental factors such as smoking are considered to contribute to its development and recurrence [7]. Among hospitalized COVID-19 patients, one in every 99 patients experienced a spontaneous pneumothorax, with a higher mortality rate compared to those without COVID-19 [8]. Although the exact mechanism remains unclear, it has been observed in patients receiving ventilation or developing fibrotic changes [8].

The management and treatment of spontaneous pneumothorax in DMD patients are challenging due to the absence of specific guidelines. Treatment strategies depend on pneumothorax size, symptoms, and etiology, aiming to evacuate air, close air leaks, and prevent recurrence [7]. For asymptomatic patients with a small (<20%) unilateral pneumothorax, simple observation is appropriate, while recommended interventions for larger spontaneous pneumothorax include simple aspiration, pleural catheter placement, drainage, chemical pleurodesis, and surgical treatment via thoracoscopy if chemical pleurodesis failed as an adjunct to prevent recurrence [7,9]. Managing spontaneous pneumothorax in DMD patients involves avoiding lung recruitment strategies and minimizing positive pressure ventilation from non-invasive ventilation to prevent further deterioration and worsening of pneumothorax [10,11].

Pulmonary function tests play a crucial role in monitoring respiratory function in both ambulatory and non-ambulatory DMD patients. Starting at the age of 5-6 years, these tests are performed annually or biannually for ambulatory patients and every six months for non-ambulatory DMD patients [1,7]. Supportive care remains the primary management approach for patients with DMD, including nocturnal NIPPV for those with hypoventilation or hypercapnia symptoms [1]. Despite being rare, spontaneous pneumothorax can occur as a complication of non-invasive ventilation in patients with neuromuscular diseases like DMD.

## Conclusions

This case highlights the potential complications, including spontaneous pneumothorax, in patients with COVID-19, particularly those with underlying conditions such as DMD. These patients face an increased risk of mortality, emphasizing the need for careful evaluation and cautious measures, including the identification of pleural blebs or bullae and careful NIPPV use. Further research and investigation are necessary to enhance our understanding and refine treatment strategies for managing pneumothorax in this specific patient population.
